# Dark Antibacterial Activity of Rose Bengal

**DOI:** 10.3390/ijms20133196

**Published:** 2019-06-29

**Authors:** Faina Nakonechny, Margarita Barel, Arad David, Simor Koretz, Boris Litvak, Elena Ragozin, Ariel Etinger, Oz Livne, Yosef Pinhasi, Gary Gellerman, Marina Nisnevitch

**Affiliations:** 1 Department of Chemical Engineering, Biotechnology and Materials, Ariel University, Ariel 4070000, Israel; 2 Department of Electrical and Electronics Engineering, Ariel University, Ariel 4070000, Israel; 3 Department of Chemical Sciences, Ariel University, Ariel 4070000, Israel

**Keywords:** Rose Bengal, *S. aureus*, ultrasonic excitation, radio wave activation, silicon, immobilization

## Abstract

The global spread of bacterial resistance to antibiotics promotes a search for alternative approaches to eradication of pathogenic bacteria. One alternative is using photosensitizers for inhibition of Gram-positive and Gram-negative bacteria under illumination. Due to low penetration of visible light into tissues, applications of photosensitizers are currently limited to treatment of superficial local infections. Excitation of photosensitizers in the dark can be applied to overcome this problem. In the present work, dark antibacterial activity of the photosensitizer Rose Bengal alone and in combination with antibiotics was studied. The minimum inhibitory concentrations (MIC) value of Rose Bengal against *S. aureus* dropped in the presence of sub-MIC concentrations of ciprofloxacin, levofloxacin, methicillin, and gentamicin. Free Rose Bengal at sub-MIC concentrations can be excited in the dark by ultrasound at 38 kHz. Rose Bengal immobilized onto silicon showed good antibacterial activity in the dark under ultrasonic activation, probably because of Rose Bengal leaching from the polymer during the treatment. Exposure of bacteria to Rose Bengal in the dark under irradiation by electromagnetic radio frequency waves in the 9 to 12 GHz range caused a decrease in the bacterial concentration, presumably due to resonant absorption of electromagnetic energy, its transformation into heat and subsequent excitation of Rose Bengal.

## 1. Introduction

The global growth and spread of bacterial resistance to antibiotics obligates a search for new approaches to eradication of pathogenic bacteria. Photodynamic antimicrobial chemotherapy (PACT) shows very promising prospects for becoming an alternative to antibiotic treatment. PACT is based on the activation of photosensitizers (PSs), which are compounds with a developed system of conjugated double bonds. Illumination of PSs by visible light leads to energy transfer from the excited PS molecules to dissolved molecular oxygen. This leads to the formation of reactive oxygen species, which cause irreversible damage to the bacterial cells that ends in the cells’ death [1,2,3,4]. PACT is known as a powerful means for killing Gram-positive and Gram-negative bacteria when applied alone or in combination with antibiotics [5,6,7,8,9,10,11]. However, due to low penetration of visible light into tissues, its applications are currently limited to treatment of superficial local infections [12]. Various methods of PS excitation in the dark can be applied in order to overcome this problem. In our previous works we showed that PSs can be activated by chemiluminescent light generated by a chemiluminescent reaction of luminol in the absence of external illumination [13,14,15]. This method was shown to be efficient against Gram-positive *S. aureus* and Gram-negative *E. coli* when methylene blue was used as the PS. An alternative approach to eradication of bacteria by PSs in the dark is based on their activation by ultrasound. We [16] and others [17,18,19,20] demonstrated that microbial cells can be inactivated by PSs under 20–40 kHz ultrasonic treatment. Ultrasonic activation exhibits good prospects for treatment of inner infections, since ultrasound can be focused on the required site, thus activating only PS molecules found in the infected region. In this case the PS molecules actually act as sonosensitizers.

Immobilization of PSs onto a solid phase pushes the boundaries of PS application, including their continuous use and reuse. It was reported that PSs can be immobilized onto carriers such as polystyrene [21,22], polycarbonate, poly(methyl methacrylate) [22], polyethylene, and polypropylene [23]. In all of these cases, immobilized PSs showed high antibacterial activity under illumination by an external source of visible light. Activation of immobilized PSs in the dark can be performed by ultrasonic treatment.

Another way to activate PSs in the dark may be by implementation of nonionizing radio frequency electromagnetic waves. It was recently reported that radio waves at a frequency of 27 MHz can be applied for activation of Si-based nanosensitizers [24]. Transferrin [25] and fullerene [26] were proposed for treatment of cancer, when the radiosensitizers were activated at a frequency of 13.56 MHz. To the best of our knowledge, sensitization of PSs by radio waves and their antibacterial application has not been reported to date.

**Figure 1:**
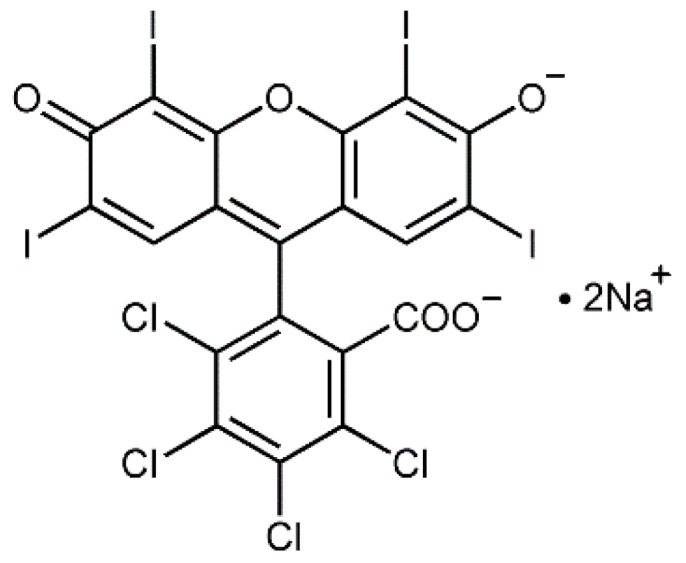
Structural formula of Rose Bengal.

In our work we studied antibacterial properties of Rose Bengal, which is known as an efficient antibacterial PS. Rose Bengal is a water-soluble PS with a characteristic purplish-red color and an absorbance spectrum with λ_max_ at 546 nm [27]. The structural formula of Rose Bengal is presented at Figure 1.

The high efficiency of Rose Bengal against bacteria under illumination is well-known [11,28,29,30]. The aims of the present work are to study the dark antibacterial activity of Rose Bengal alone and when combined with antibiotics, to test excitation of free and immobilized Rose Bengal by ultrasound and to examine possible Rose Bengal activation by radio waves in the dark for further application against internal infections.

## 2. Results and Discussion

### 2.1. Dark Activity of Rose Bengal

PSs exhibit high antimicrobial activity under illumination. However, at high concentrations they are known to possess a certain dark activity and to inactivate cells in the dark. The dark activity of Rose Bengal on several bacteria was described by Bond et al. [31], Hsieh et al. [32], Shrestha et al. [33], Coulson et al. [34], Nakoniecna et al. [35], and Goulart et al. [36], although the reason for this phenomenom is not clear [37]. In the present work we examined the dark activity of Rose Bengal alone and in the presence of several antibiotics against *S. aureus* by detection of minimum inhibitory concentrations (MIC). In our experiments, MIC values of Rose Bengal and the antibiotics ciprofloxacin hydrochloride, levofloxacin, gentamicin sulfate and methicillin were determined under dark conditions. The MIC values of Rose Bengal in the presence of sub-MIC concentrations of each antibiotic were then determined. In all cases, the sub-MIC concentrations of each antibacterial agent were chosen as half of the MIC-value. The results of this experiment are presented in Table 1. The MIC of Rose Bengal in the dark is 0.125 mg/mL, which is 200-fold higher than the value we measured previously under illumination [11]. Addition of antibiotics caused a 4-fold decrease in the dark MIC of Rose Bengal in the presence of ciprofloxacin and levofloxacin, a 2-fold decrease in the presence of methicillin and more than a 100-fold decrease in the presence of gentamicin. Such significant reductions in MIC values indicate a synergistic effect between the action of Rose Bengal and the antibiotics. This observation is consistent with an analogous effect reported earlier for Rose Bengal combined with antibiotics under illumination where MIC values of antibiotics decreased upon the addition of sub-MIC concentrations of Rose Bengal [9,10,11].

A combination of Rose Bengal and gentamicin caused not only a drastic drop in the MIC of the former in the presence of the latter (Table 1). Rather, the addition of Rose Bengal at a concentration much below the dark MIC value (0.008 mg/mL) led to a decrease in the gentamicin MIC from 0.005 to 0.0016 mg/mL. In order to examine the effect of gentamicin at the sub-MIC concentration on the cell functioning, growth curves of *S. aureus* in the absence and in the presence of MIC and sub-MIC concentrations of gentamicin were obtained. Growth of bacteria in the presence of the sub-MIC concentration of gentamicin resulted in a 12% decrease in the growth rate and a 15% decrease in the final OD_660_ of the cell suspension. However, these differences were not significant, since the *p*-values were higher than 0.7. In contradistinction, the growth rate in the presence of the MIC concentration of gentamicin dropped by more than two-fold and the final OD_660_ decreased by 86% (*p*-value = 0.026). It can be concluded that when gentamicin is applied alone at the sub-MIC concentration, it does not cause any significant stress to the cells, contrary to its application at the MIC concentration. The peculiar effect of the Rose Bengal and gentamicin combination can be explained by cooperation of the two antimicrobials. Gentamicin belongs to a group of aminoglycosides, which are broad-spectrum antibiotics whose action is based on protein synthesis inhibition [38]. The process of gentamicin penetration into a bacterial cell depends on the permeability of the bacterial membrane and energetic factors [39]. Since Rose Bengal is known to affect bacterial membrane components [40], its application undoubtedly facillitates penetration of gentamicin into *S. aureus* cells. In addition, a bacterial transmembranous negative electrical potential is considered as a driving force for aminoglycoside entrance into a cell [41]. Addition of Rose Bengal, which is negatively charged under physiological conditions (Figure 1), probably increases the membrane potential and stimulates gentamicin uptake, thus causing pronounced cell eradication.

### 2.2. Ultrasonic Activation of Free and Immobilized Rose Bengal

**Figure 2:**
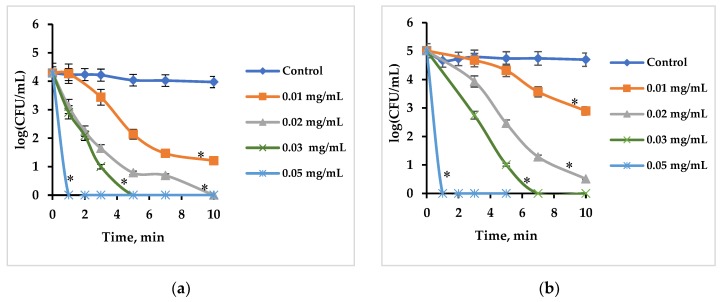
Effect of free Rose Bengal at various concentrations in the dark under ultrasonic excitation on *S. aureus* at initial concentrations of (**a**) 10^4^ and (**b**) 10^5^ CFU/mL. Control—*S. aureus* cells treated by ultrasound only. Error bars present standard deviations. The asterisk (*) denotes statistically significant differences with the control (*p* < 0.05).

After examination of dark activity, the possibility of exciting Rose Bengal by ultrasound in the dark was examined when Rose Bengal was applied in a free form or immobilized onto a polymer. In the free form, the antibacterial activity of Rose Bengal depended on its concentration and on the initial bacterial cell concentration. Figure 2 shows that the Rose Bengal concentration is critical for its activity: at high concentrations Rose Bengal caused very profound cell inhibition after short sonication periods, whereas at low concentrations Rose Bengal did not eradicate all *S. aureus* cells even after 10 min of sonication. When the initial bacterial cell concentration was increased from 10^4^ to 10^5^ CFU/mL, it was necessary to either apply higher concentrations of Rose Bengal or increase the sonication time in order to achieve a good antibacterial effect. At a Rose Bengal concentration of 0.03 mg/mL, *S. aureus* cells at an initial concentration of 10^4^ CFU/mL were destroyed within 5 min (Figure 2a), but, at a concentration of 10^5^ CFU/mL and the same Rose Bengal concentration, full eradication of the cells was observed only after 7 min (Figure 2b). Increasing the Rose Bengal concentration to 0.05 mg/mL led to total killing of cells already after 1 min at both initial cell concentrations (Figure 2a and b).

**Figure 3:**
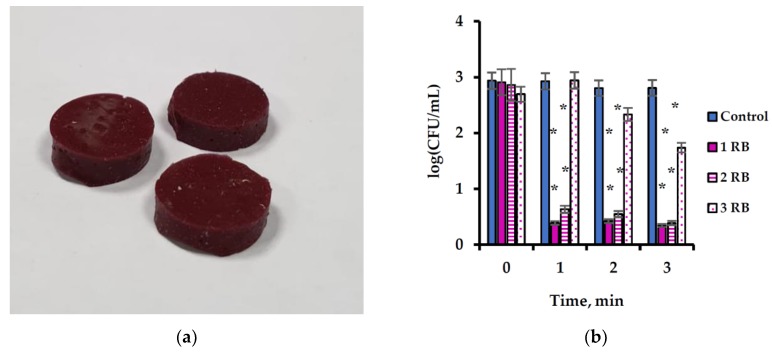
Photo of silicon tablets with immobilized Rose Bengal (**a**) and its antibacterial activity against *S. aureus* under ultrasonic activation in repeated applications (**b**). Designations: 1 RB—1^st^ application of Rose Bengal; 2 RB—2^nd^ application; and 3 RB—3^rd^ application. Control—*S. aureus* cells treated by ultrasound only. Error bars present standard deviations. The asterisk (*) denotes statistically significant differences with the control (*p* < 0.05).

Since there are very limited possibilities for application of free PSs, Rose Bengal was immobilized onto silicon at a loading of 5% (*w*/*w*) by adding Rose Bengal powder to silicon components during the polymerization stage. No covalent bonds were formed between Rose Bengal and silicon. However, since the former was homogeneously distributed in the latter, the obtained silicon tablets were evenly colored by the characteristic Rose Bengal color (Figure 3a). The tablets exhibited good antibacterial activity when applied against *S. aureus* cells in the dark under ultrasonic activation, and the cell concentration dropped by 2.5 log_10_ already after 1 min (Figure 3b). A repeated use of the same tablets yielded practically the same results. However, the tablets were almost inactive in the 3^rd^ use, and the bacterial concentration decreased by only one order of magnitude after 3 min of sonication (Figure 3b).

After the treatment, the cell suspensions appeared pink, which indicated leaching of Rose Bengal from the silicon support. The rate of leaching under sonication was as twice as high as with no sonication. Such enhancement of Rose Bengal leaching cannot be explained by heating of the sample during the sonication, since the procedure was performed for short periods of time and the temperature did not exceed 30 °C in any of the experiments.

**Figure 4:**
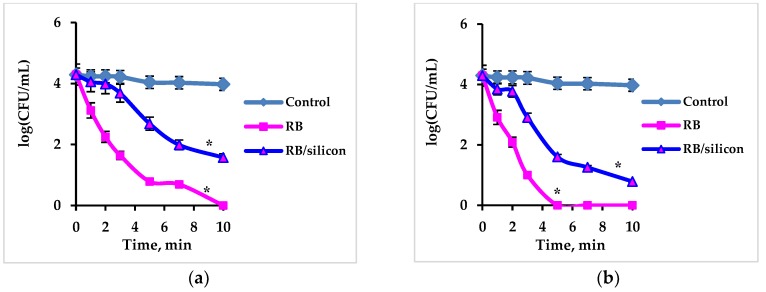

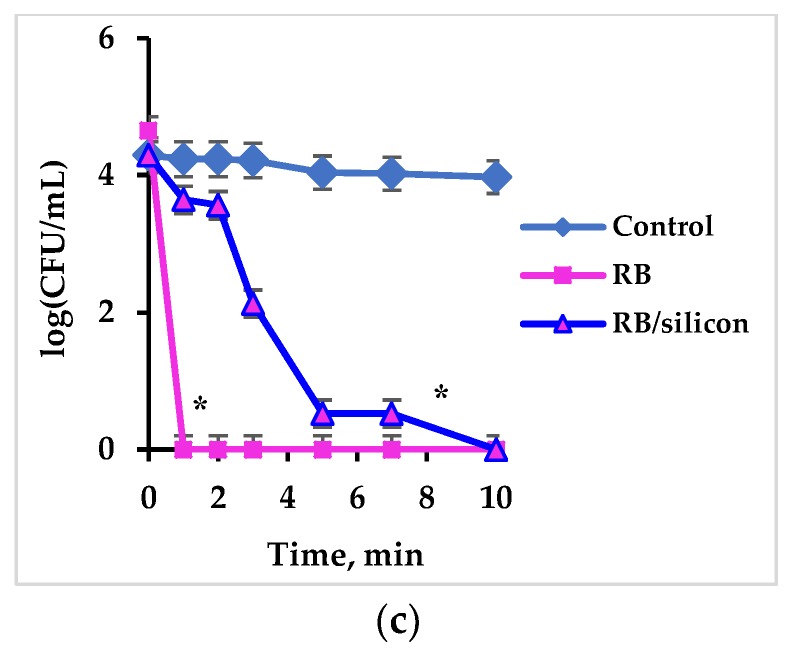
Comparison between the antibacterial activity of free Rose Bengal (designated RB) at concentrations of (**a**) 0.020 ± 0.001 mg/mL, (**b**) 0.030 ± 0.005 mg/mL, and (**c**) 0.050 ± 0.003 mg/mL with immobilized Rose Bengal (designated RB/silicon) when the concentrations of the released Rose Bengal at the end of the experiment reached: (**a**) 0.020 ± 0.004 mg/mL, (**b**) 0.030 ± 0.006 mg/mL, and (**c**) 0.050 ± 0.007 mg/mL under ultrasonic treatment in the dark. Initial concentration of *S. aureus* was 2 × 10^4^ CFU/mL. Control cells were treated by ultrasound only. Error bars present standard deviations. The asterisk (*) denotes statistically significant differences with the control (*p* < 0.05).

The phenomenon of Rose Bengal leaching raised the question of whether *S. aureus* inactivation was due to the Rose Bengal immobilized onto silicon or to the Rose Bengal which leached from the polymer into the aqueous phase. To study this issue, a series of experiments was performed in which the Rose Bengal concentration was measured in each sample, in addition to testing the bacterial concentration. These results were compared to the antibacterial activity of free Rose Bengal (Figure 4). Rose Bengal release fluctuated from one experiment to another. We therefore matched the data on the free Rose Bengal activity to the data on the immobilized Rose Bengal and compared between samples with the same Rose Bengal concentration at the end of the experiment (Figure 4). Such matching was naturally not absolutely accurate, since the Rose Bengal concentration in the samples with free Rose Bengal was constant during the course of the experiment, whereas in the samples with immobilized Rose Bengal, its concentration grew during the experiment. Nonetheless, this comparison enabled us to understand which of the factors was responsible for the bacterial eradication. Figure 4 presents a comparison between the activity of several concentrations of free and immobilized Rose Bengal, when concentrations of the released Rose Bengal reached the same values by the end of the experiment. In each case, the rate of bacterial eradication was correlated with the concentration of released Rose Bengal: when Rose Bengal release was low (0.02 mg/mL, Figure 4a, and 0.03 mg/mL, Figure 4b), total eradication of the bacteria was not achieved even after 10 min sonication, whereas when Rose Bengal release was high (final concentration of 0.05 mg/mL, Figure 4c), the cells were eradicated after this period of time. In each case, the activity of free Rose Bengal was higher than that of immobilized Rose Bengal (Figure 4a–c). This result was anticipated, since there was no Rose Bengal in the aqueous phase at the beginning of each experiment, and its concentration increased over the course of the experiment. The above findings support the assumption that the antibacterial activity of immobilized Rose Bengal was due to the continuous leaching of Rose Bengal from the solid state during the ultrasonic processing. This assumption can also explain the results shown in the Figure 3b. After the first several applications, Rose Bengal apparently undergoes massive leaching from the external silicone layers, while diffusion of Rose Bengal from deeper layers is limited. The antibacterial activity of the composite is therefore reduced already after several applications. Despite this limitation, Rose Bengal immobilized in silicone may be applied during implantation of silicone implants, since the main bacterial contamination occurs during the surgery before sewing the incisions [42,43], and the implants are treated by antibacterial agents, usually antibiotics. If the external layer of the implant will be manufactured from silicone with immobilized Rose Bengal, this could enhance antibacterial protection of the implants, enable a reduction in the applied antibiotic doses, and provide protection against resistant bacterial strains. Moreover, leaching of Rose Bengal after sewing the incisions may prevent possible postsurgery infections.

One possible mechanism of PS activation by ultrasound can be emission of sonoluminescent light, causing excitation of the PS. Sonoluminescence is a well-known phenomenon accompanying ultrasonic processing of liquids. The spectrum of maximal emittance of sonoluminescent light for an aqueous medium is between 250 and 600 nm [44], which corresponds well to the absorption spectrum of Rose Bengal that is in the range of 480 to 580 nm [16]. This fact explains our previous observation that Rose Bengal was active against Gram-positive and Gram-negative bacteria, whereas methylene blue whose absorption spectrum does not coincide with the sonoluminescent emission spectrum was inactive against the same bacteria [16]. Another mechanism of sonodynamic excitation may be based on the presence of the free radicals OH and H, generated as a result of water pyrolysis or chemical activation of sonosensitizers with formation of sensitizer-derived free radicals [45,46,47]. Further studies are needed to clarify which of the mechanisms actually takes place.

### 2.3. Activation of Rose Bengal by Radio Waves

**Figure 5:**
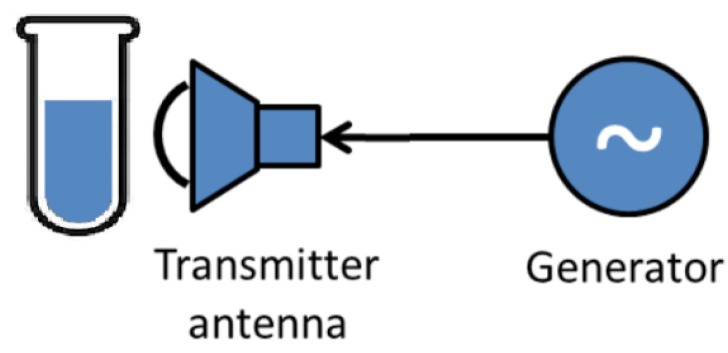
Scheme of the device for irradiation of the bacterial culture with radio frequency electromagnetic waves.

**Figure 6:**
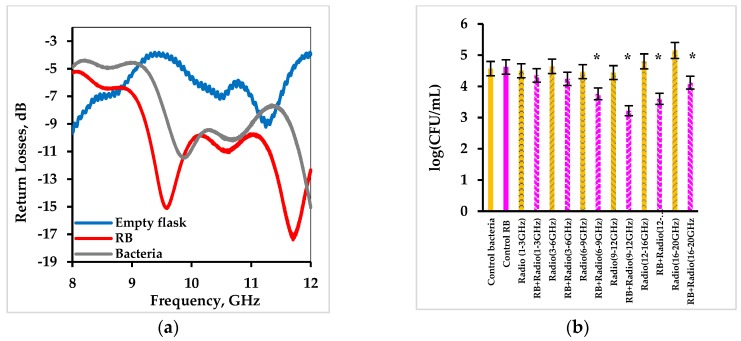
Absorption of radio waves by an empty glass flask, an aqueous solution of Rose Bengal at a concentration of 0.01 mg/mL and a suspension of *S. aureus* cells at a concentration of 4.4 × 10^4^ CFU/mL (**a**) and the effect of Rose Bengal at a concentration of 0.01 mg/mL under activation by radio waves in the dark in the range of 1–20 GHz on eradication of *S. aureus* at an initial cell concentration of 4.4 × 10^4^ CFU/mL (**b**). Error bars present standard deviations. The asterisk (*) denotes statistically significant differences with the control (*p* < 0.05).

Experiments on activation of Rose Bengal by radio waves were performed using the scheme shown in Figure 5. The system consists of a generator of radio waves in the frequency range of 1 to 20 GHz and a pyramidal horn antenna that transmitted the radiation towards test-tubes containing bacterial suspensions in the presence and absence of Rose Bengal. Since electromagnetic waves in this frequency regime cannot be absorbed by molecules and noting that radiation in the radio frequencies is not ionizing, it was important to test whether resonant absorption of radio wave energy by system components took place at the applied radiation frequencies. Return losses of radio waves were therefore measured in the system. Figure 6a shows that the empty glass flask practically did not absorb radio waves, whereas an aqueous solution of Rose Bengal had peaks of resonance absorption at 9.6 and 11.7 GHz and a suspension of *S. aureus* cells in saline had peaks at 9.9 GHz and ca. 12 GHz.

In order to understand whether absorption of radio wave energy can affect Rose Bengal activation, cells of *S. aureus* alone and in the presence of Rose Bengal were irradiated with radio waves at different frequencies. Dark control series included *S. aureus* cells in saline and cells in the presence of Rose Bengal but not subjected to radio waves.

There was no decrease in cell concentration in control untreated cells or in the presence of Rose Bengal (dark controls) as well as in control series where the cells were treated with radio waves in the absence of Rose Bengal (Figure 6b). In contradistinction, treatment of cells by radio waves in the presence of Rose Bengal led to a decrease in the bacterial concentration. The most significant drop of 1.5 log_10_ was observed in the frequency range of 9 to 12 GHz (Figure 6b). Since this range matches the resonance frequencies of the Rose Bengal solution and the cell suspension, the system was studied further, and the frequency range corresponding to the highest effect was detailed in each subsequent experiment.

**Figure 7:**
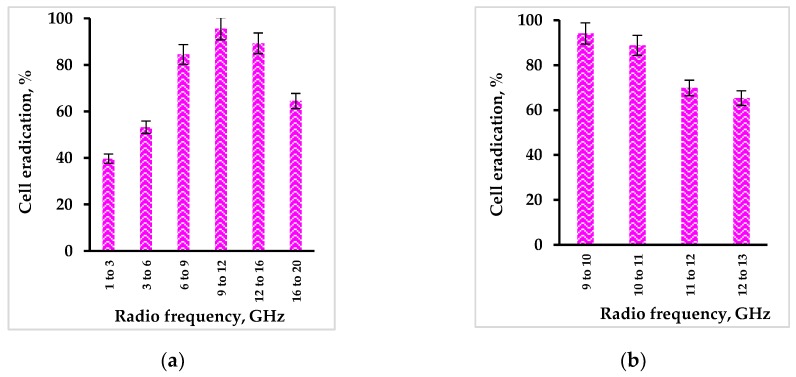

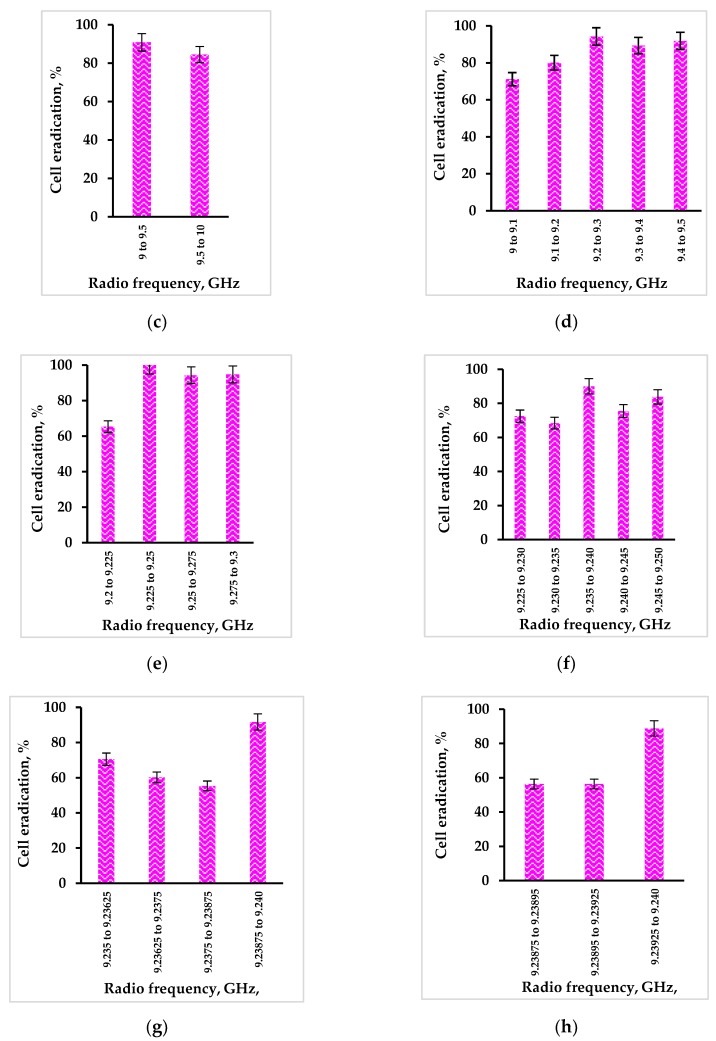
Effect of Rose Bengal at a concentration of 0.01 mg/mL under activation by radio waves at different frequency ranges on eradication of *S. aureus* at an initial cell concentration of 4.4.10^4^ CFU/mL in the dark: (**a**) 1–20 GHz, (**b**) 9–13 GHz, (**c**) 9–10 GHz, (**d**) 9–9.5 GHz, (**e**) 9.2–9.3 GHz, (**f**) 9.225–9.25 GHz, (**g**) 9.235–9.24 GHz, and (**h**) 9.23875–9.24 GHz. Error bars present standard deviations.

The experiments included testing of live cells in the following order; first, in the range of 1 to 20 GHz (Figure 6b and Figure 7a), then 9–13 GHz (Figure 7b), after that 9–10 GHz (Figure 7c), 9–9.5 GHz (Figure 7d), 9.2–9.3 GHz (Figure 7e), 9.225–9.25 GHz (Figure 7f), 9.235–9.24 GHz (Figure 7g) and, finally, 9.23875–9.24 GHz (Figure 7h). Control experiments were performed in all series and showed no effect on *S. aureus* cells with and without addition of Rose Bengal in the dark and none under radio frequency wave treatment in the absence of Rose Bengal.

The data presented in Figure 7 show that treatment of *S. aureus* by radio wave frequencies in the range of 9 to 12 GHz in the presence of Rose Bengal caused the highest cell eradication (Figure 7a). Further detailing of radio wave frequencies did not lead to any increase in the antibacterial effect. Since resonant absorbance of radio waves by Rose Bengal and by cells was observed exactly in this range (Figure 6a), this was probably the reason for the best effect of radio waves on activation of Rose Bengal and its antibacterial effect.

The most probable mechanism of Rose Bengal excitation is transformation of electromagnetic energy into heat, causing activation of Rose Bengal followed by transfer of energy to dissolved oxygen, i.e., Rose Bengal behaved like a radiosensitizer. Tamarov et al. [24] and Chung et al. [25] believe that radiosensitizers act via heat activation due to hyperthermia caused by dissipation of electromagnetic energy, leading to thermal injury of malignant cells. For this reason, radiosensitizers can actually be referred to as thermosensitizers [25]. In our study, exposure of cells to radio waves did not cause any increase in temperature, since irradiation was low power, but the resonance absorption of radio wave energy could cause transformation of locally-absorbed electromagnetic energy into heat with further excitation of Rose Bengal. Excitation of Rose Bengal in the dark mediated by radio waves may result in possible practical applications for curing internal infections at sites where visible light cannot permeate and not only for topical infections under illumination, as currently applied.

## 3. Materials and Methods

### 3.1. Materials and Bacterial Strains

Rose Bengal (80%) was purchased from Sigma-Aldrich Chemie GmbH (Schnelldorf, Germany). The antibiotics ciprofloxacin hydrochloride (98%), levofloxacin (98%), and methicillin (98%) were purchased from Alfa Aesar (USA). Gentamicin sulfate (>99%) was purchased from Formedium Ltd. (England). Silicon RTVI (A-42) (>92%) and a solidifier, Silicon RTVI (B-42) (>92%), were purchased from ELGAD, Israel. Growth media Brain Heart broth (BH) and Brain Heart agar (BHA) were purchased from Acumedia (USA) and Antibiotic medium 3 for MIC determination studies was purchased from Becton Dickinson & Co. (Le Pont de Claix, France). Methicillin-sensitive *S. aureus* strain ATCC 11541 was purchased from ATCC (Manassas, VA, USA).

### 3.2. Immobilization of Rose Bengal onto Silicon

One gram of polymerization component A (milky transparent silicon RTVI, A-42) was placed in a beaker and 0.125 g of component B (solidifier—Silicon RTVI, B-42) were added to the same beaker. Immediately after addition of component B, 0.05 g of Rose Bengal was added and the viscous solution was mixed carefully with a glass stick for 1 min, placed in a Petri plate, and left overnight at room temperature to obtain, after polymerization, a round silicon rubber with approximately 1 cm thickness.

The obtained dry silicon rubber was washed 3 times with 70% aqueous ethanol in order to remove remains of Rose Bengal which were not captured inside the silicone. It was then cut into 1 cm diameter circles which were ready for use in further experiments.

### 3.3. Bacterial Growth

*S. aureus* bacteria were grown as described in [27]. In brief, cultures of *S. aureus* were grown on BHA, then transferred into BH, grown overnight at 37 °C and shaking at a 170 rpm and diluted with the Antibiotic medium 3 or BH to obtain an OD_600_ = 0.1, which corresponded to an initial cell concentration of 10^8^ CFU/mL. The obtained cell suspension was used for further experiments.

### 3.4. Examination of Dark Rose Bengal Activity in the Presence of Antibiotics

#### 3.4.1. MIC Determination of Antibiotics

The MIC of the antibiotics ciprofloxacin hydrochloride, levofloxacin, gentamicin sulfate and methicillin were determined by a standard broth double dilutions procedure [48]. In brief, the antibiotic in 2 mL of antibiotic medium was distributed by a double dilution method into a series of tubes. The microbial suspension was then added to the tubes to a final concentration of 10^6^ CFU/mL. The tubes were incubated for 24 h at 37 °C under dark conditions, and their turbidity was checked. The antibiotic concentration in the last transparent tube in the series, which corresponded to the minimal antibiotic concentration inhibiting bacterial growth, was defined as the MIC value.

#### 3.4.2. MIC Determination of Rose Bengal in the Presence of Sub-MIC Antibiotic Concentrations

A series of tubes with 2 mL of double dilutions of Rose Bengal in an antibiotic medium was prepared. Antibiotic was added to each tube up to a sub-MIC concentration defined as half of the MIC-value for each antibiotic. A bacterial suspension was added to each tube to a final concentration of 10^6^ CFU/mL. The tubes were then treated as described in Section 3.4.1.

### 3.5. Ultrasonic Activation of Free and Silicon-Immobilized Rose Bengal

Bacteria were grown as described in Section 3.3 to a concentration of 10^8^ CFU/mL and then diluted with sterile saline by serial decimal dilutions to concentrations of 10^3–^10^7^ CFU/mL. Three milliliters of bacterial suspensions was transferred into flat-bottom 2.5 cm diameter 20 mL vials. Before the experiment, samples were taken from each vial to determine the initial bacterial concentration. Free Rose Bengal at various concentrations or Rose Bengal immobilized onto silicone (one 1-cm circle, Section 3.2, per vial) was added to all vials, except for the controls. After adding Rose Bengal, all subsequent procedures were performed under dark conditions. Vials with bacterial suspensions with or without Rose Bengal were held tight to the bottom of an ultrasonic bath in a plastic holder VU03H (SMEG, Italy) and treated by ultrasound at a frequency of 38 kHz and a field strength of 4.1 W/cm^3^ for 1 to 10 min as described in [16]. After the treatment, 100 µL samples were diluted by several decimal dilutions and were spread over BHA plates with a Drigalsky spreader. The plates were incubated at 37 °C overnight and the bacterial cell concentration was determined taking dilutions into account using the viable count method, where CFU were counted by means of a colony counter Scan 500 (Interscience, Saint Nom la Bretèche, France). In control experiments, bacterial cultures were tested in the absence of Rose Bengal without sonication, in the absence of Rose Bengal under sonication and in the presence of Rose Bengal without sonication.

### 3.6. Activation of Free Rose Bengal by Radio Waves

Bacteria were grown as described in Section 3.5 and then diluted with sterile saline to 10^4^ CFU/mL. Ten milliliters of the bacterial suspension was distributed into 15 mL test tubes. One-hundred microliters portions of the Rose Bengal stock solution (1 mg/mL) were added to the tubes to obtain a concentration of 0.01 mg/mL. After adding Rose Bengal, all subsequent procedures were performed under dark conditions. The tubes were exposed to radio waves of various frequencies for half an hour, using a radiation system based on the N5173B EXG Analog Signal Generator (Keysight Technologies, CA, USA). The variable frequency oscillator was tuned within a range of frequencies from 1 up to 20 GHz and produced a radio frequency power of 40 mW (16 dBm) fed to a broadband horn antenna JXTXLB-10180 (A-info) gaining a directivity of 11 dBi (typ). In the range of 9 to 12 GHz, a pyramidal horn antenna with a rectangular cross section and a directivity of 15 dBi was used. The radiation intensity irradiating the tube was 1.8 mW/cm^2^. At the end of the experiments, bacterial concentration was determined by live count as described in Section 3.5. In control experiments, bacterial cultures were tested in the presence and absence of Rose Bengal without exposure to radio frequency waves and in the absence of Rose Bengal under exposure to radio waves.

### 3.7. Statistical Analysis

The results obtained from at least three independent experiments carried out in duplicates were analyzed by single-factor ANOVA analyses. The difference between the results was considered significant when the *p*-value was less than 0.05. Quantitative results are presented as the mean *±* standard error.

## 4. Conclusions

Rose Bengal possesses dark antibacterial activity which is enhanced upon combination with antibiotics. The MIC value of Rose Bengal against *S. aureus* drops in the presence of sub-MIC concentrations of ciprofloxacin hydrochloride, levofloxacin, methicillin, and gentamicin sulfate. Free Rose Bengal at sub-MIC concentrations can be excited in the dark by ultrasound at 38 kHz. Rose Bengal immobilized onto silicon shows good antibacterial activity in the dark under ultrasonic activation, probably because of Rose Bengal leaching from the polymer during the treatment. Exposure of bacteria to electromagnetic radio frequency waves in the 9 to 12 GHz range in the presence of Rose Bengal in the dark caused a decrease in the bacterial concentration, presumably due to resonant absorption of electromagnetic energy, its transformation into heat and subsequent excitation of Rose Bengal. Excitation of Rose Bengal in the dark mediated by ultrasound and radio waves may result in possible practical applications for curing internal infections at sites where visible light cannot permeate.

## Figures and Tables

**Table 1:** MIC values of Rose Bengal and antibiotics for *S. aureus* in the dark.

Antimicrobial	MIC ^1^, mg/mL	MIC ^1^ of Rose Bengal, mg/mL, in the Presence of Sub-MIC of Antibiotics
Rose Bengal	0.125 ± 0.001	NR ^2^
Ciprofloxacin hydrochloride	0.025 ± 0.001	0.0312 ± 0.0006
Levofloxacin	0.025 ± 0.001	0.0312 ± 0.006
Gentamicin sulfate	0.005 ± 0.001	0.0011 ± 0.0001
Methicillin	0.0025 ± 0.0008	0.0625 ± 0.0013

^1^ Average ± SE; ^2^ NR – not relevant.

## Abbreviations

PACT	Photodynamic antimicrobial chemotherapy
PS	photosensitizer
MIC	minimum inhibitory concentration
SE	Standard error
CFU	Colony forming units
BH	Brain Heart broth
BHA	Brain Heart agar
OD_660_	Optical density at 660 nm
